# Edge valency-based entropies of tetrahedral sheets of clay minerals

**DOI:** 10.1371/journal.pone.0288931

**Published:** 2023-07-21

**Authors:** Yong Tang, Muhammad Labba, Muhammad Kamran Jamil, Muhammad Azeem, Xiujun Zhang

**Affiliations:** 1 School of Computer Science, Chengdu University, Chengdu, China; 2 Department of Mathematics, Riphah International University Lahore, Lahore, Pakistan; Shiv Nadar University, INDIA

## Abstract

Humanity has always benefited from an intercapillary study in the quantification of natural occurrences in mathematics and other pure scientific fields. Graph theory was extremely helpful to other studies, particularly in the applied sciences. Specifically, in chemistry, graph theory made a significant contribution. For this, a transformation is required to create a graph representing a chemical network or structure, where the vertices of the graph represent the atoms in the chemical compound and the edges represent the bonds between the atoms. The quantity of edges that are incident to a vertex determines its valency (or degree) in a graph. The degree of uncertainty in a system is measured by the entropy of a probability. This idea is heavily grounded in statistical reasoning. It is primarily utilized for graphs that correspond to chemical structures. The development of some novel edge-weighted based entropies that correspond to valency-based topological indices is made possible by this research. Then these compositions are applied to clay mineral tetrahedral sheets. Since they have been in use for so long, corresponding indices are thought to be the most effective methods for quantifying chemical graphs. This article develops multiple edge degree-based entropies that correlate to the indices and determines how to modify them in order to assess the significance of each type.

## Introduction

The entropy of a probability measures the uncertainty of a system. This concept is strongly based on statistical methodology. It is mainly used for chemical structures and their corresponding graphs. It also provides information about graph structure and chemical topologies. It was used as a notion for the first time in 1955. In many scientific and technical fields, entropy has applications. Intrinsic and extrinsic entries are determined in this way. The idea of degree power is used to investigate networks as information functional. The authors put forward the idea of entropy for different topological indices. The entropy of probability distributions is the foundation described in [1, 2]. This parameter imparts a lot of information about structures, graphs, and chemical topology of networks in network theory, by which a parameter is known as degree powers. It is a base for graph theory in applied mathematics to investigate networks’ information function. Authors put forward the idea of entropy for a variety of networks like Shannon used for the entropy of probability distribution.

In modeling and designing any chemical network, can be converted into a graph, and this theory plays a very important role. By using the graph’s theoretical transformation of a chemical network or structure, we can determine many properties, like physicochemical properties, thermodynamical properties, and biological activities. Among them, by using the topological indices, certain properties can be characterized. Edge-weighted base entropy is also from the family of topological indices.

A simple molecular finite graph in chemical graph theory denotes both atoms and chemical bonds in terms of vertices and edges, respectively. The numeric value of the topological index shows the physical, chemical, and topological properties of a graph. With the help of chemical graph theory, we can model the mathematical phenomenon of chemical networks. It is related to the non-trivial use of graph theory to solve a molecular problem. This theory plays a vital role in the field of chemical science. Chem-informatics is the combination of chemistry, mathematics, and information science. Using QSAR and QSPR, which are examined by chem-informative [3], we can predict bioactivities and physical-chemical properties of a chemical compound.

The authors of [4] computed some topological indices and corresponding entropies. They discussed the relationship between numerical and graphical on the basis of topological indices and their entropies. Furthermore, they also discussed the topological indices that give fruitful results for the structural properties of *g* − *C*_3_*N*_4_, and some related applications are also available. According to [5], the authors find the entropy value by applying different parameters like the total number of vertices, edges, and degrees of any graph. Then they compared the results with different graphs. They resulted that increasing the vertices and edges of graphs, affects in increasing the entropy of such graphs. Some crystal structures based on non-kekulean benzenoid sub-structures are discussed in terms of entropies by using the degree-based topological indices [6]. They also computed the relationship between degree-based topological indices and degree-based entropy. They provided some applications in different topics like chemical, biological and physical reactivity processes. Given in the article [7], topological indices of *si*2*c*3 − *I* and *si*2*c*3 − *II* and based on the results entropy measures are discussed. Here in [8], the researchers contributed to a tool for molecular graphs that was based on the weight of edges, known as entropy. They related the entropy measure to polynomial functions. Particularly, they computed the entropy measure of the magnesium iodide structure and found different entropies, like Zagreb and atom bond entropies.

In [9], authors discussed the benzenoid hydrocarbon chemical structure. Shannon applied the benzenoid entropy to the transmission rates of telephonic channels, optical communication, and wireless. In this paper, the author discusses the characteristics and effects of graph entropies in different topological indices like coronoid polycyclic aromatic hydrocarbons. They computed the entropies through topological indices on degree terminal vertices. In [10], researchers find the effective roll of metal-insulator transition superlattices (GST-Sw) with different topological properties. Particularly, they discussed the atom-bond degree-based topological indices that are applied in the heat formation of single crystal and monolayered structure of Ge-sb-te. Researchers of [11], find different entropies of crystallography chemical networks. The author particularly computed hyper and augmented Zagreb, forgotten, and Balaban entropies for the crystallography structures of cup-rite *cu*_2_*o* and titanium difluoride *t*_*i*_*f*_2_ by using the different topological tools. First, second, modified and augmented Zagreb indices, symmetric division, harmonic, inverse sum index, and forgotten indices are computed in [12]. They computed these indices for two chemical structures: crystal cubic carbon and carbon graphite networks.

The author in [13] derived some degree-based temperature descriptors. He also computed the temperature entropies of molecular structure and related them to degree-based temperature with the help of specific information functions. He also compared graphs with calculated functions. All the computed results from the given information provided more effective drug and covid-19 vaccines. The authors of [14] focused on the carbon nanotubes that are useful in tissue engineering and investigated the various entropies. They computed some entropies for different variants of carbon nanotubes. They also investigated and compared the results of armchair carbon nanotubes with other forms of structure. The researchers of [15], investigated the relation of natural polymer of cellulose network and pharmacological applicant which provided the good result in a different structure. They computed different K-Banhatti indices and their entropies. The nature polymer of cellulose networks and their entropies are effective in various topics of chemical structural theory. Structures of Polycyclic aromatic hydrocarbons are discussed in [16], authors elaborated intriguing properties based on the graph-theoretical parameters. They discussed entropy measures and their numerical values for given structures. Different entropies of the molecular structure of HCQ are discussed in [17] by using degrees of vertices and edges. Particularly, they computed degree-based topological characteristics of hydroxyethyl starch conjugated structures with hydroxychloroquine. They also presented some relations of different entropies with other structures. In the end, they compared the proven results in terms of numerical and graphical form.

In [18], the main purpose of this article is to study the properties of graphs and then discuss the structure of hyaluronic acid (HA) curcumin conjugates. Moreover, the author computed the entropies by using the degree-based topological indices with the help of the information function. Indices are linked with total *π*-electron property and measured some entropies of trans-PD-(*NH*_2_)s lattice and metal-organic super-lattice structures, by using the different graph parameters, in [19]. Structures of dendrimers based on cyclotriphosphazene (*N*_3_*P*_3_) are applied to the topics of balanced and computed the EPR temperature spectrum, discussed in [20]. First of all, the author computed eccentricity-based indices and their entropies. After the above indices result, the author presented it numerically and graphically. Entropies of some variants of Y-shaped nano-junctions are discussed in [21]. By using topological indices, knowledge discovery and representations of molecular structures are considered in [22]. Particularly, authors computed topological indices like atom-bond connectivity (ABC), the fourth version of ABC, geometric arithmetic (GA), and the fifth version of GA. Finally, the authors computed the above indices for octa-nano sheets, equilateral triangular sheets, tetra sheets, rectangular sheets, and rectangular tetra sheets.

Graph entropies of porous graphene chemical networks are found in [23], with the use of topological indices. They also provide some application of chosen indices-based entropies, particularly for the porous graphene structure. Different topological indices-based entropies of armchair carbon nanotubes are found in [24]. They provide some applications of variants of carbon nanotubes, like capped, uncapped, and semi-capped ones, which are purely used in memory devices and tissue engineering. Authors of [25] introduced the self-powered vertex degree topological indices and computed the graph entropy measures for tessellations networks, such as the isentropic later which is known as a non-isomorphic tessellation of kekulenes. Researchers in [26] computed the entropies of the structures of fractal and Cayley tree-type dendrimers by using the different topological indices and their degree-based results. The chosen structures are types of dendrimers denoted by *Fr* and *Cm*, *n*.

The Mostar, Padmakar-Ivan (PI), and Szeged indices of the molecular graph of two kinds of dendrimers, in which the Phthalocyanines and Porphyrine are discussed in [27]. The author also computed the entropies of these structures and relates these with other topological indices and their entropies.

Three classes of isoreticular metal-organic frameworks, graph entropies, enumeration of circuits, walks and topological properties are computed and analyzed in [28]. They also used different properties like eccentricities, radius, diameter, vertex, and the degrees to find the thermochemistry for the isoreticular network. In the [29] article, the authors describe and explain the characterization of two-dimensional coronene fractals in terms of different topological indices. The author also computed the entropy measure and compared it with other topological indices. They used machine learning techniques for robust computation of enthalpies. Moreover, they used NMR and ESR spectroscopic patterns of coronene fractals. In [30], authors determine the data difficulty handling by using the entropy measure. For this, they collected data from different fields of science, but for this research work they faced a problem: the data is not accurate, and for this, they used the entropy measure to get the non-redundant, non-correlated data. The authors computed a good solution by using good algorithms for research and entropy measures. There are 25 entropy measures that is taken for the classification procedure and compared to its result. The crystal structure of the polyphenylene network for photocatalysis is discussed in [31], and some topological indices are found. By using these indices, they work for the thermodynamic properties, namely entropy and heat, which yield beneficial results for the crystal structure. They concluded that effective application resulted from the chosen topological indices and their corresponding entropies.

A biochemistry network namely t-level hypertrees of corona product of hypertrees with path are produced in [32]. The author also works with different topological indices, like eccentricity-based indices and their entropies for the chosen biochemical network. Crystal structure of titanium difluoride *T*_*i*_*F*_2_ and crystallographic structure of *CU*_2_*O* discussed in the paper given by [11]. The author also computed the different entropies of these structures and relates them to different topological indices like, the first, second, and third redefined Zagreb indices, the fourth atom bond connectivity index, the fifth geometric arithmetic index, and the Sanskruti index, and their entropies are discussed. For more recent research work on topological indices [33–39].

## Methodology of proposed work

Let *E*(*G*) be the edge set and *V*(*G*) be the vertex set of a graph. The degree of a vertex *v* in a graph is determine by the attached edges with the vertex in the graph, and it is denoted by *d*_*v*_. A distance *d*(*a*, *b*) between *a* and *b* vertices are the shortest length path of graph. The order and size of the graph are |*V*(*G*)| and |*E*(*G*)|, respectively. Following are some formulations of the topological indices we used to develop the entropies.

- **General Randić index** [40, 41]
  $$\begin{array}{c} R_{\alpha }\left(G\right)=\sum_{ab\in E(G)}{\left(d_{a}d_{b}\right)}^{\alpha }. \end{array}$$ (1)
- **General sum-connectivity index** [40, 41]
  $$\begin{array}{c} SCI\left(G\right)=\sum_{ab\in E(G)}{\left(d_{a}+d_{b}\right)}^{\alpha }. \end{array}$$ (2)
- **Generalized Zagreb index** [40, 41]
  $$\begin{array}{c} Z_{\alpha }\left(G\right)=\sum_{ab\in E(G)}(d_{a}^{\alpha }d_{b}^{\beta }+d_{b}^{\alpha }d_{a}^{\beta }). \end{array}$$ (3)
- **Fourth Zagreb index** [40, 41]
  $$\begin{array}{c} Z_{4}\left(G\right)=\sum_{ab\in E(G)}d_{a}\left(d_{a}+d_{b}\right). \end{array}$$ (4)
- **Fifth Zagreb index** [40, 41]
  $$\begin{array}{c} Z_{5}\left(G\right)=\sum_{ab\in E(G)}d_{b}\left(d_{a}+d_{b}\right). \end{array}$$ (5)
- **Geometric-arithmetic index** [40, 41]
  $$\begin{array}{c} GA\left(G\right)=\sum_{ab\in E(G)}\frac{2\sqrt{d_{a}d_{b}}}{d_{a}+d_{b}}. \end{array}$$ (6)
- **Atom-bond connectivity index** [40, 41]
  $$\begin{array}{c} ABC\left(G\right)=\sum_{ab\in E(G)}\sqrt{\frac{d_{a}+d_{b}-2}{d_{a}d_{b}}}. \end{array}$$ (7)
- **Harmonic index** [40, 41]
  $$\begin{array}{c} H\left(G\right)=\sum_{ab\in E(G)}\frac{2}{d_{a}+d_{b}}. \end{array}$$ (8)
- **Variation of Randić index** [40, 41]
  $$\begin{array}{c} R^{\prime }\left(G\right)=\sum_{ab\in E(G)}\frac{1}{\text{max}\{d_{a},d_{b}\}}. \end{array}$$ (9)
- **Symmetric division degree index** [40, 41]
  $$\begin{array}{c} SDD\left(G\right)=\sum_{ab\in E(G)}\frac{d_{a}^{2}+d_{b}^{2}}{d_{a}d_{b}}. \end{array}$$ (10)

The first part of our methodology is given above, corresponding to Eqs 1 to 10; the equations given below are edge-weighted entropies. Some of these entropies are developed already, but most of them are novel and we are developing and computing for the first time. Entropy is denoted by the letter $\lambda_{R_{\alpha }}\left(G\right)$ for the topological index *R*_*α*_ for the graph *G*.

- **Entropy of gerenal Randić index** [42, 43]
  $$\begin{array}{c} \lambda_{R_{\alpha }}\left(G\right)=-\frac{1}{R_{\alpha }\left(G\right)}\text{log}[\prod_{ab\in E(G)}{\left\{{\left(d_{a}d_{b}\right)}^{\alpha }\right\}}^{{\left(d_{a}d_{b}\right)}^{\alpha }}]+\text{log}\;(R_{\alpha }\left(G\right)). \end{array}$$ (11)
- **Entropy of gerenal Sum-connectivity index** [42, 43]
  $$\begin{array}{c} \lambda_{SC}\left(G\right)=-\frac{1}{SC(G)}\text{log}[\prod_{ab\in E(G)}{\left\{{\left(d_{a}+d_{b}\right)}^{\alpha }\right\}}^{{\left(d_{a}+d_{b}\right)}^{\alpha }}]+\text{log}\;(SC(G)). \end{array}$$ (12)
- **Entropy of gerenalized Zagreb index** [42, 43]
  $$\begin{array}{c} \lambda_{Z_{\alpha }}\left(G\right)=-\frac{1}{Z_{\alpha }\left(G\right)}\text{log}[\prod_{ab\in E(G)}{\left(d_{a}^{\alpha }d_{b}^{\beta }+d_{b}^{\alpha }d_{a}^{\beta }\right)}^{\left(d_{a}^{\alpha }d_{b}^{\beta }+d_{b}^{\alpha }d_{a}^{\beta }\right)}]+\text{log}\;(Z_{\alpha }\left(G\right)). \end{array}$$ (13)
- **Entropy of fourth Zagreb index** [42, 43]
  $$\begin{array}{c} \lambda_{Z_{4}}\left(G\right)=-\frac{1}{Z_{4}\left(G\right)}\text{log}[\prod_{ab\in E(G)}{\left\{d_{a}(d_{a}+d_{b}\right)\}}^{\left[d_{a}(d_{a}+d_{b})\right]}]+\text{log}\;(Z_{4}\left(G\right)). \end{array}$$ (14)
- **Entropy of fifth Zagreb index** [42, 43]
  $$\begin{array}{c} \lambda_{Z_{5}}\left(G\right)=-\frac{1}{Z_{5}\left(G\right)}\text{log}[\prod_{ab\in E(G)}{\left\{d_{b}(d_{a}+d_{b}\right)\}}^{\left[d_{b}(d_{a}+d_{b})\right]}]+\text{log}\;(Z_{5}\left(G\right)). \end{array}$$ (15)
- **Entropy of geometric-arithmetic index** [42, 43]
  $$\begin{array}{c} \lambda_{GA}\left(G\right)=-\frac{1}{GA(G)}\text{log}[\prod_{ab\in E(G)}{\left(\frac{2\sqrt{d_{a}.d_{b}}}{d_{a}+d_{b}}\right)}^{\frac{2\sqrt{d_{a}.d_{b}}}{d_{a}+d_{b}}}]+\text{log}\;(GA(G)). \end{array}$$ (16)
- **Entropy of atom-bond connectivity index** [42, 43]
  $$\begin{array}{c} \lambda_{ABC}\left(G\right)=-\frac{1}{ABC(G)}\text{log}[\prod_{ab\in E(G)}{\left(\frac{\sqrt{d_{a}+d_{b}-2}}{d_{a}d_{b}}\right)}^{\frac{\sqrt{d_{a}+d_{b}-2}}{d_{a}d_{b}}}]+\text{log}\;(ABC(G)). \end{array}$$ (17)
- **Entropy for Harmonic index** [42, 43]
  $$\begin{array}{c} \lambda_{H}\left(G\right)=-\frac{1}{HI(G)}\text{log}[\prod_{ab\in E(G)}{\left(\frac{2}{d_{a}+d_{b}}\right)}^{\frac{2}{d_{a}+d_{b}}}]+\text{log}\;(H(G)). \end{array}$$ (18)
- **Entropy for Variation of Randić index** [42, 43]
  $$\begin{array}{c} \lambda_{R^{\prime }}\left(G\right)=-\frac{1}{R^{\prime }\left(G\right)}\text{log}[\prod_{ab\in E(G)}{\left(\frac{1}{max\{d_{a},d_{b}\}}\right)}^{\frac{1}{\text{max}\{d_{a},d_{b}\}}}]+\text{log}\;(R^{\prime }\left(G\right)). \end{array}$$ (19)
- **Entropy of symmetric division degree index** [42, 43]
  $$\begin{array}{c} \lambda_{SDD}\left(G\right)=-\frac{1}{SDD(G)}\text{log}[\prod_{ab\in E(G)}{\left(\frac{d_{a}^{2}+d_{b}^{2}}{d_{a}d_{b}}\right)}^{\frac{d_{a}^{2}+d_{b}^{2}}{d_{a}d_{b}}}]+\text{log}\;(SDD(G)). \end{array}$$ (20)

In this work, we introduced some topological based entropy measures, like Entropy of gerenal Randić index, Entropy of gerenal Sum-connectivity index, Entropy of gerenalized Zagreb index, Entropy of fourth Zagreb index, Entropy of fifth Zagreb index, Entropy of geometric-arithmetic index, Entropy of atom-bond connectivity index, Entropy for Harmonic index, Entropy for Variation of Randić index, and Entropy of symmetric division degree index. At the end, we took a chemical network clay mineral tetrahedral sheets to evaluate the performance of introduced entropy measures.

## Chemical structure for the computational work

**Fig 1:**
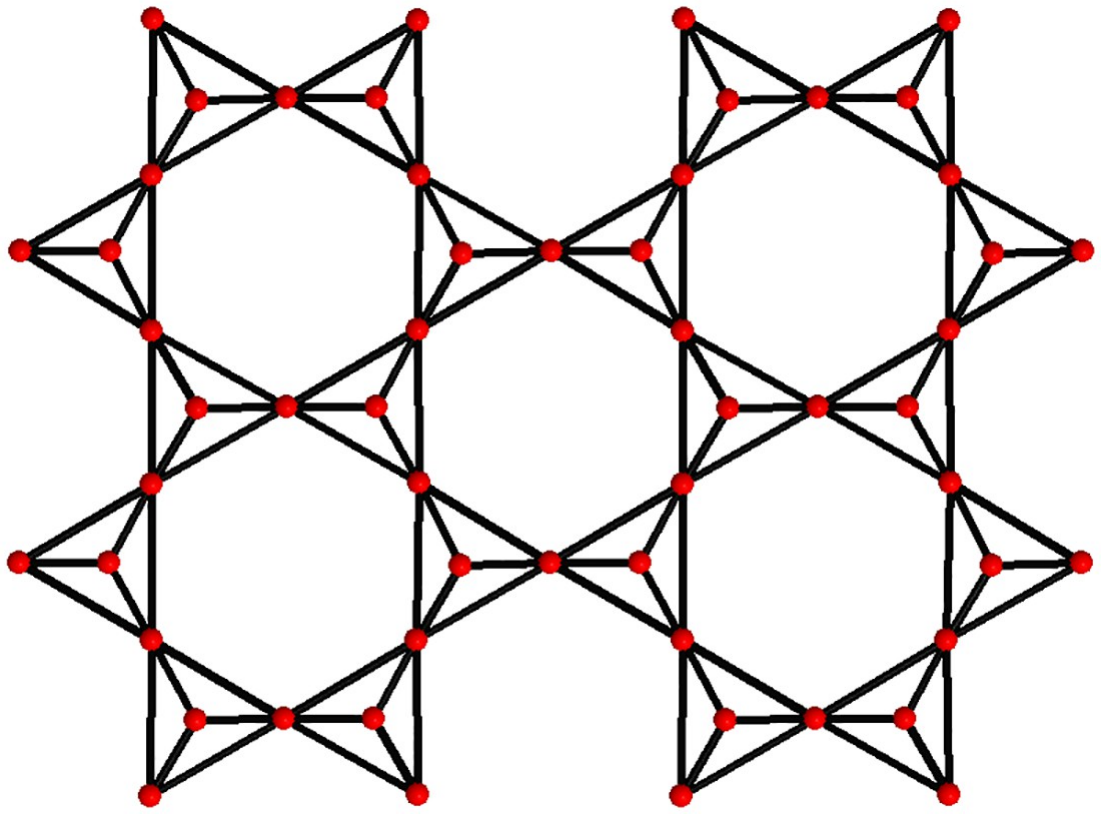
A particular example of tetrahedral sheets of clay mineral.

Clay mineral tetrahedral sheets are represented by the notation *TSCM*_*β*,*γ*_. Various edge degree-based entropy measurements are applied to tetrahedral sheets in the aforementioned paper. One of the platonic graphs, the tetrahedral graph, has four vertices and six edges. It can be seen as a solid. The only planer construction of its isomorphic graphs, such as the complete graph *K*_4_ and wheel graph *W*_4_, is the tetrahedral graph. Chemical graph theory uses tetrahedral sheets made by tetrahedral graph polymerization to reflect silicone and other clay minerals. On tetrahedral sheets, interdisciplinary topics can be accommodated via combinatorial properties such as labeling, coloring, enumeration, and indexing, as well as algorithmic operations such as shortest path and spanning tree. Fig 1 depicts the tetrahedral sheets of clay minerals *TSCM*_*β*,*γ*_ for *β* = 2, *γ* = 2. There are topological indices available in [44] for Eqs 21 through 30.

The graph *TSCM*_*β*,*γ*_ contains 10*βγ* + 7*β* + *γ* are vertices and 24*βγ* + 12*β* total count of edges. In *TSCM*_*β*,*γ*_ there are two types of vertices with degree 3 or 6. The vertex set of *TSCM*_*β*,*γ*_ can be distributed according to their degrees.

Let

$$\begin{array}{c} V_{i}=\left\{a\in V\left(TSCM_{\beta ,\gamma }\right):d_{a}=i\right\}. \end{array}$$

This indicates that the vertices of degree *i* are present in the set *V*_*i*_. According to their degree, the set of vertices is as follows:

$$\begin{array}{c} V_{3}=\left\{a\in V\left(TSCM_{\beta ,\gamma }\right):d_{a}=3\right\}\\ V_{6}=\left\{a\in V\left(TSCM_{\beta ,\gamma }\right):d_{a}=6\right\} \end{array}$$

Since |*V*_3_| = 4*βγ* + 6*β* + 2*γ* and |*V*_3_| = 6*βγ* + *β* − *γ*, we can also subdivide the edges of *TSCM*_*β*,*γ*_ into following three subsets according to the degree of its end vertices.

$$\begin{array}{c} E_{3,3}=\left\{ab\in TSCM_{\beta ,\gamma }:d_{a}=3,d_{b}=3\right\}\\ E_{3,6}=\left\{ab\in TSCM_{\beta ,\gamma }:d_{a}=3,d_{b}=6\right\}\\ E_{6,6}=\left\{ab\in TSCM_{\beta ,\gamma }:d_{a}=6,d_{b}=6\right\} \end{array}$$

Note that *E*(*TSCM*_*β*,*γ*_) = *E*_3,3_ ∪ *E*_3,6_ ∪ *E*_*β*,*γ*_. The number of edges incident to one vertex of degree 3 and other vertices are 3, 6 are 4*β* + 2*γ*, 12*βγ* + 10*β* + 2*γ* respectively, so |*E*_3,3_| = 4*β* + 2*γ*, |*E*_3,3_| = 12*βγ* + 10*β* + 2*γ*. The edges that are incident to two vertices of degree 6 are now the remaining number of edges, which are |*E*_6,6_| = 12*βγ* − 2*β* − 4*γ*.

## Main results and some computational work

In this section, we will present our key findings and computational work pertaining to topological indices and entropies.

$$\begin{array}{c} R_{\alpha }(G)=12\beta \gamma \left({\left(18\right)}^{\alpha }+{\left(36\right)}^{\alpha }\right)+\beta (\left(4\right){\left(9\right)}^{\alpha }+10{\left(18\right)}^{\alpha }-2{\left(36\right)}^{\alpha })+\gamma (2{\left(9\right)}^{\alpha }+2{\left(18\right)}^{\alpha }-4{\left(36\right)}^{\alpha }). \end{array}$$ (21)

$$\begin{array}{c} SC(G)=12\beta \gamma ({\left(9\right)}^{\alpha }+{\left(12\right)}^{\alpha })+\beta (4{\left(6\right)}^{\alpha }+10{\left(9\right)}^{\alpha }-2{\left(12\right)}^{\alpha })+\gamma ({\left(2\right)}^{\alpha }+2{\left(9\right)}^{\alpha }-4{\left(12\right)}^{\alpha }). \end{array}$$ (22)

$$\begin{array}{cc} Z_{\alpha }(G)=\left({\left(3\right)}^{\alpha }.{\left(3\right)}^{\beta }+{\left(3\right)}^{\beta }.{\left(3\right)}^{\alpha }\right)\left(4\beta +2\gamma \right)\left({\left(3\right)}^{\alpha }.{\left(6\right)}^{\beta }+{\left(3\right)}^{\beta }.{\left(6\right)}^{\alpha }\right){\left(12\beta \gamma +10\beta +2\gamma \right)(\left(6\right)}^{\alpha }.{\left(6\right)}^{\beta }\\ & +{\left(6\right)}^{\beta }.{\left(6\right)}^{\alpha })\left(12\beta \gamma -2\beta -4\gamma \right). \end{array}$$ (23)

$$\begin{array}{c} Z_{4}(G)=18(4\beta +2\gamma )+27(12\beta \gamma +10\beta +2\gamma )+72(12\beta -2\beta -4\gamma ). \end{array}$$ (24)

$$\begin{array}{c} R_{5}(G)=18(4\beta +2\gamma )+54(12\beta \gamma +10\beta +2\gamma )+72(12\beta -2\beta -4\gamma ). \end{array}$$ (25)

$$\begin{array}{c} GA(G)=4\beta \gamma \left(2\sqrt{2}+3\right)+\frac{2\beta }{3}(3+10\sqrt{2})+\frac{2\gamma }{3}(2\sqrt{2}-3). \end{array}$$ (26)

$$\begin{array}{cc} ABC(G)= & 2\beta \gamma \left(\sqrt{14}+\sqrt{10}\right)+\frac{\beta }{3}\left(8+5\sqrt{14}\right)+\frac{\gamma }{3}\left(4+4\sqrt{14}-2\sqrt{10}\right). \end{array}$$ (27)

$$\begin{array}{cc} H(G)= & \frac{14}{3}\beta \gamma +\frac{29}{9}\beta +\frac{4}{9}\gamma . \end{array}$$ (28)

$$\begin{array}{cc} R^{\prime }(G)= & 54\beta \gamma +29\beta +\gamma . \end{array}$$ (29)

$$\begin{array}{cc} SDD(G)= & 10\beta \gamma +7\beta +\gamma . \end{array}$$ (30)

Now discuss the following properties of a general topological invariant (*IG*) based on the degree of vertices in a graph (*G*).

- **Entropy of general Randić index**
$$\begin{array}{c} \lambda_{R_{\alpha }}(G)=-\frac{1}{R_{\alpha }\left(G\right)}\text{log}\left[\prod_{ab}\in E\left(G\right){\left[{\left[d_{a}d_{b}\right]}^{\alpha }\right]}^{{\left[d_{a}d_{b}\right]}^{\alpha }}\right]+\text{log}\;R_{\alpha }\left(G\right) \end{array}$$
  Computing the general Randić index by using Eq 1, we will get the result given in Eq 21. Now using Eq 21 in the formula of entropy of general Randić index, which is given in Eq 11. After simplification, we will get the result given in Eq 31. Putting the value in entropy
  $$\begin{array}{cc} \lambda_{R_{\alpha }}(G)= & -\frac{1}{12\beta \gamma \left({\left(18\right)}^{\alpha }+{\left(36\right)}^{\alpha }\right)+\beta \left(4\right){\left(9\right)}^{\alpha }+10{\left(18\right)}^{\alpha }-2{\left(36\right)}^{\alpha }+\gamma \left[2{\left(9\right)}^{\alpha }+2{\left(18\right)}^{\alpha }-4{\left(36\right)}^{\alpha }\right]}\\ & \text{log}\left[{\left[{\left(9^{\alpha }\right)}^{\left(9^{\alpha }\right)}\right]}^{\left(4\beta +2\gamma \right)}\cdot {\left[{\left(18^{\alpha }\right)}^{\left(18^{\alpha }\right)}\right]}^{\left(12\beta \gamma +10\beta +2\gamma \right)}\cdot {\left[{\left(36^{\alpha }\right)}^{\left(36^{\alpha }\right)}\right]}^{\left(12\beta \gamma -2\beta -4\gamma \right)}\right]\\ & +\text{log}[12\beta \gamma \times \left({\left(18\right)}^{\alpha }+{\left(36\right)}^{\alpha }\right)+\beta \left(4\right){\left(9\right)}^{\alpha }+10{\left(18\right)}^{\alpha }-2{\left(36\right)}^{\alpha }+\gamma [2{\left(9\right)}^{\alpha }+2{\left(18\right)}^{\alpha }-4{\left(36\right)}^{\alpha }]]. \end{array}$$ (31)
- **Entropy of Gerenal Sum-connectivity index**
$$\begin{array}{c} \lambda_{SC}\left(G\right)=-\frac{1}{SCI\left(G\right)}\text{log}\left[\prod_{ab\in E\left(G\right)}{\left[{\left[d_{a}+d_{b}\right]}^{\alpha }\right]}^{{\left[d_{a}+d_{b}\right]}^{\alpha }}\right]+\text{log}\left(SCI\left(G\right)\right). \end{array}$$
  Using Eq 2 to compute the general sum-connectivity index yields the result provided in Eq 22. Now using Eq 22 in the formula for the entropy of the general sum-connectivity index, which is given in Eq 12. After simplification, we will get the result given in Eq 32. Putting the of entropy of the sum-connectivity index
  $$\begin{array}{cc} \lambda_{SCI}(G)= & -\frac{1}{12\beta \gamma \left[{\left(9\right)}^{\alpha }+{\left(12\right)}^{\alpha }\right]+\beta \left[4{\left(6\right)}^{\alpha }+10{\left(9\right)}^{\alpha }-2{\left(12\right)}^{\alpha }\right]+\gamma \left[{\left(2\right)}^{\alpha }+2{\left(9\right)}^{\alpha }-4{\left(12\right)}^{\alpha }\right]}\\ & \text{log}\left[{\left[{\left(6^{\alpha }\right)}^{\left(6^{\alpha }\right)}\right]}^{\left(4\beta +2\gamma \right)}\cdot {\left[{\left(9^{\alpha }\right)}^{\left(9^{\alpha }\right)}\right]}^{\left(12\beta \gamma +10\beta +2\gamma \right)}\cdot {\left[{\left(36^{\alpha }\right)}^{\left(36^{\alpha }\right)}\right]}^{\left(12\beta \gamma -2\beta -4\gamma \right)}\right]\\ & +\text{log}\;(12\beta \gamma \left({\left(9\right)}^{\alpha }+{\left(12\right)}^{\alpha }\right)+\beta \left(4\right){\left(6\right)}^{\alpha }+10{\left(9\right)}^{\alpha }-2{\left(12\right)}^{\alpha }+\gamma \left[2{\left(6\right)}^{\alpha }+2{\left(9\right)}^{\alpha }-4{\left(12\right)}^{\alpha }\right]). \end{array}$$ (32)
- **Entropy of generalized Zagreb index**
$$\begin{array}{c} \lambda_{Z_{\alpha }}(G)=-\frac{1}{Z_{\alpha }\left(G\right)}\text{log}{\left[\prod_{ab\in E(G)}\left(d_{a}^{\alpha }d_{b}^{\beta }+d_{b}^{\alpha }d_{a}^{\beta }\right)\right]}^{\left(d_{a}^{\alpha }d_{b}^{\beta }+d_{b}^{\alpha }d_{a}^{\beta }\right)}+\text{log}\left(Z_{\alpha }\left(G\right)\right). \end{array}$$
  Using Eq 3 to compute the generalized Zagreb index, we obtain the result provided in Eq 23. Now include Eq 23 into the calculation for the entropy of the generalized Zagreb index, which is given in Eq 13. After simplification, we will get the result given in Eq 33. Putting the value of the entropy generalized Zagreb index
  $$\begin{array}{cc} \lambda_{Z_{\alpha }}(G)= & -\frac{1}{\left({\left(3\right)}^{\alpha }\times {\left(3\right)}^{\beta }+{\left(3\right)}^{\beta }\times {\left(3\right)}^{\alpha }\right)\left(4\beta +2\gamma \right)\left({\left(3\right)}^{\alpha }\times {\left(6\right)}^{\beta }+{\left(3\right)}^{\beta }\times {\left(6\right)}^{\alpha }\right)\left(12\beta \gamma +10\beta +2\gamma \right)}\\ & \times \frac{1}{({\left(6\right)}^{\alpha }\times {\left(6\right)}^{\beta }+{\left(6\right)}^{\beta }\times {\left(6\right)}^{\alpha })\left(12\beta \gamma -2\beta -4\gamma \right)}\\ & \times \text{log}\left[{\left(18^{\alpha }\beta \right)}^{\left(18^{\alpha }\beta \right)\left(4\beta +2\gamma \right)}\times {\left[{\left(36^{\alpha }\beta \right)}^{\left(36^{\alpha }\beta \right)}\right]}^{\left(12\beta \gamma +10\beta +2\gamma \right)}\times {\left[{\left(72^{\alpha }\beta \right)}^{\left(72^{\alpha }\beta \right)}\right]}^{\left(12\beta \gamma -2\beta -4\gamma \right)}\right]\\ & +\text{log}[\left({\left(3\right)}^{\alpha }\times {\left(3\right)}^{\beta }+{\left(3\right)}^{\beta }\times {\left(3\right)}^{\alpha }\right)\left(4\beta +2\gamma \right)\left({\left(3\right)}^{\alpha }\times {\left(6\right)}^{\beta }+{\left(3\right)}^{\beta }\times {\left(6\right)}^{\alpha }\right)\\ & \left(12\beta \gamma +10\beta +2\gamma \right)({\left(6\right)}^{\alpha }\times {\left(6\right)}^{\beta }+{\left(6\right)}^{\beta }\times {\left(6\right)}^{\alpha })\left(12\beta \gamma -2\beta -4\gamma \right)] \end{array}$$ (33)
- **Entropy of fourth Zagreb index**
$$\begin{array}{c} \lambda_{Z_{4}}(G)=-\frac{1}{Z_{4}\left(G\right)}\text{log}[\prod_{ab\in E(G)}\sum_{ab\in E(G)}d_{a}{\left(d_{a}+d_{b}\right)}^{d_{a}\left(d_{a}+d_{b}\right)}]+\text{log}\left(Z_{4}\left(G\right)\right). \end{array}$$
  Using Eq 4 to compute the fourth Zagreb index yields the answer provided in Eq 24. Now include Eq 24 into the calculation for the entropy of the fourth Zagreb index, which is given in Eq 14. After simplification, we will get the result given in Eq 34. Putting the values of entropy of the Fourth Zagreb index
  $$\begin{array}{cc} \lambda_{Z_{4}}(G)= & -\frac{1}{18(4\beta +2\gamma )+27(12\beta \gamma +10\beta +2\gamma )+72(12\beta -2\beta -4\gamma )}\\ & \times \text{log}[{\left({\left(18\right)}^{\left(18\right)}\right)}^{\left(4\beta +2\gamma \right)}\times {\left({\left(27\right)}^{\left(27\right)}\right)}^{\left(12\beta \gamma +10\beta +2\gamma \right)}\times {\left({\left(72\right)}^{\left(72\right)}\right)}^{\left(12\beta \gamma -2\beta -4\gamma \right)}]. \end{array}$$ (34)
- **Entropy of fifth Zagreb index**
$$\begin{array}{c} \lambda_{Z_{5}}(G)=-\frac{1}{Z_{5}\left(G\right)}\text{log}[\prod_{ab\in E(G)}\;\sum_{ab\in E(G)}d_{b}{\left(d_{a}+d_{b}\right)}^{d_{b}\left(d_{a}+d_{b}\right)}]+\text{log}\left(Z_{5}\left(G\right)\right). \end{array}$$
  Using Eq 5 to get the fifth Zagreb index yields the answer provided in Eq 25. Using Eq 25 in the calculation for the entropy of the Fifth Zagreb index, which is given in Eq 15, is the next step. After simplification, the solution will be given by Eq 35.
  $$\begin{array}{cc} \lambda_{Z_{5}}(G)= & -\frac{1}{18(4\beta +2\gamma )+54(12\beta \gamma +10\beta +2\gamma )+72(12\beta -2\beta -4\gamma )}log[{\left({\left(18\right)}^{\left(18\right)}\right)}^{\left(4\beta +2\gamma \right)}\\ & \times {\left({\left(54\right)}^{\left(54\right)}\right)}^{\left(12\beta \gamma +10\beta +2\gamma \right)}\times {\left({\left(72\right)}^{\left(72\right)}\right)}^{\left(12\beta \gamma -2\beta -4\gamma \right)}]. \end{array}$$ (35)
- **Entropy of Geometric-Arithmetic index**
$$\begin{array}{c} \lambda_{GA}\left(G\right)=-\frac{1}{GA\left(G\right)}\text{log}\left[\prod_{ab\in E\left(G\right)}{\left(\frac{2\sqrt{d_{a}.d_{b}}}{d_{a}+d_{b}}\right)}^{\left(\frac{2\sqrt{d_{a}.d_{b}}}{d_{a}+d_{b}}\right)}\right]+\text{log}\left(GA\left(G\right)\right). \end{array}$$
  Using Eq 6 to compute the Geometric-Arithmetic index yields the result provided in Eq 26. Using Eq 26 in Eq 16’s entropy formula for the Geometric-Arithmetic index, the entropy is calculated. After simplification, the solution will be given by Eq 36. Putting the values entropy of the geometric-arithmetic index
  $$\begin{array}{cc} \lambda_{GA}(G)= & -\frac{1}{4\beta \gamma \left(2\sqrt{2}+3\right)+\frac{2\beta }{3}\left[3+10\sqrt{2}\right]+\frac{2\gamma }{3}\left[2\sqrt{2}-3\right]}\text{log}[{\left({\left(\frac{2\sqrt{2}}{3}\right)}^{\frac{2\sqrt{2}}{3}}\right)}^{12\beta \gamma +10\beta +2\gamma }]\\ & +\text{log}[4\beta \gamma \left(2\sqrt{2}+3\right)+\frac{2\beta }{3}\left[3+10\sqrt{2}\right]+\frac{2\gamma }{3}\left[2\sqrt{2}-3\right]]. \end{array}$$ (36)
- **Entropy for atom-bond connectivity index**
$$\begin{array}{c} \lambda_{ABC}(G)=-\frac{1}{ABC(G)}\text{log}[\prod_{ab\in E(G)}{\left(\sqrt{\frac{d_{a}+d_{b}-2}{d_{a}d_{b}}}\right)}^{\sqrt{\frac{d_{a}+d_{b}-2}{d_{a}d_{b}}}}]+\text{log}\left(ABC(G)\right). \end{array}$$
  Calculating the Atom-Bound Connectivity index using Eq 7 yields Eq 27 as the outcome. Using Eq 27 in the calculation for the entropy of the Atom-Bound Connectivity index, which is given in Eq 17, is the next step. After simplification, the solution will be given by Eq 37. Putting the values in entropy
  $$\begin{array}{c} \lambda_{ABC}(G)=-\frac{1}{2\beta \gamma \left(\sqrt{14}+\sqrt{10}\right)+\frac{\beta }{3}\left(8+5\sqrt{14}-\sqrt{10}\right)+\frac{\gamma }{3}\left(4\sqrt{14}-2\sqrt{10}\right)}\\ \text{log}[{\left(\frac{2}{9}\right)}^{{\frac{2}{9}}^{4\beta +2\gamma }}\times {\left[{\left(\sqrt{\frac{7}{18}}\right)}^{\sqrt{\frac{7}{18}}}\right]}^{12\beta \gamma +10\beta +2\gamma }\times {\left[{\left(\sqrt{\frac{10}{6}}\right)}^{\sqrt{\frac{10}{6}}}\right]}^{12\beta \gamma -2\beta -4\gamma }]\\ \text{log}[2\beta \gamma \left(\sqrt{14}+\sqrt{10}\right)+\frac{\beta }{3}\left(8+5\sqrt{14}-\sqrt{10}\right)+\frac{\gamma }{3}\left(4\sqrt{14}-2\sqrt{10}\right)]. \end{array}$$ (37)
- **Entropy for harmonic index**
$$\begin{array}{c} \lambda_{H}(G)=-\frac{1}{H(G)}\text{log}[\prod_{ab\in E(G)}{\left[\frac{2}{d_{a}+d_{b}}\right]}^{\frac{2}{d_{a}+d_{b}}}]+\text{log}[H(G)]. \end{array}$$
  Using Eq 8 to compute the Harmonic index yields the result of Eq 28. Now utilizing Eq 28 in the formula of entropy of index, which is given in Eq 18, is the Harmonic given in Eq 18. After simplification, the solution will be given by Eq 38.
  $$\begin{array}{cc} \lambda_{H}(G)= & -\frac{1}{\frac{14}{3}\beta \gamma +\frac{29}{9}\beta +\frac{4}{9}\gamma }\text{log}[{\left(\frac{1}{3}\right)}^{{\frac{1}{3}}^{4\beta +2\gamma }}{\left(\frac{2}{3}\right)}^{{\frac{2}{3}}^{12\beta +10\beta +2\gamma }}{\left(\frac{1}{6}\right)}^{{\frac{1}{6}}^{12\beta \gamma -2\beta -4\gamma }}]\\ & +\text{log}[\frac{14}{3}\beta \gamma +\frac{29}{9}\beta +\frac{4}{9}\gamma ]. \end{array}$$ (38)
  **Entropy for symmetric division degree index**
$$\begin{array}{c} \lambda_{SDD}(G)=-\frac{1}{SDD(G)}\text{log}[\prod_{ab\in E(G)}{\left(\frac{d_{a}^{2}+d_{b}^{2}}{d_{a}d_{b}}\right)}^{\left(\frac{d_{a}^{2}+d_{b}^{2}}{d_{a}d_{b}}\right)}]+\text{log}\;(SDD(G)). \end{array}$$
  Using Eq 9 to compute the Symmetric Division Degree index yields Eq 29 as the result. Now applying Eq 29 in the calculation for the entropy of the Symmetric Division Degree index, which is given in Eq 19, yields the following result: After simplification, the solution will be given by Eq 39. Putting the values of in entropy
  $$\begin{array}{cc} \lambda_{SDD}(G)= & -\frac{1}{54\beta \gamma +29\beta +\gamma }\text{log}[{\frac{1}{3}}^{\frac{1}{3}}4\beta +2\gamma \times {\frac{1}{6}}^{12\beta \gamma +10\beta +2\gamma }{\frac{1}{6}}^{12\beta \gamma -2\beta -4\gamma }]\\ & +\text{log}[\frac{1}{54\beta \gamma +29\beta +\gamma }]. \end{array}$$
  $$\begin{array}{c} \lambda_{SDD}(G)=-\frac{1}{54\beta \gamma +29\beta +\gamma }\text{log}\;[44.54]+\text{log}\;[54\beta \gamma +29\beta +\gamma ]. \end{array}$$ (39)

## Conclusion

A network’s or structure’s edge weight-based entropy offers structural details and in-depth content in the form of mathematical equations. This study contained the edge valency-based entropies of tetrahedral sheets of clay minerals. It highlights the molecular attributes in the form of a logarithmic function and offers the structural information of chemical networks or their related build-up graphs. Since then, over 3000 topological graph indices are registered in Chemical Data Bases. This research area is studied by mathematicians and chemists. So one can introduced new versions of topological indices based entropy measures. Moreover, all the newly developed topological indices based entropy measures can be computed for different types of chemical structures and networks.
