# Correction: Edge valency-based entropies of tetrahedral sheets of clay minerals

**DOI:** 10.1371/journal.pone.0315032

**Published:** 2024-12-03

**Authors:** Yong Tang, Muhammad Labba, Muhammad Kamran Jamil, Muhammad Azeem, Xiujun Zhang

The affiliation for the fourth author is incorrect. Muhammad Azeem is not affiliated with #1 but with #2: Department of Mathematics, Riphah International University Lahore, Lahore, Pakistan.
